# Enhanced calcium ion mobilization in osteoblasts on amino group containing plasma polymer nanolayer

**DOI:** 10.1186/s13578-018-0220-8

**Published:** 2018-03-21

**Authors:** Susanne Staehlke, Henrike Rebl, Birgit Finke, Petra Mueller, Martina Gruening, J. Barbara Nebe

**Affiliations:** 1 Dept. of Cell Biology, University Medical Center Rostock, Schillingallee 69, 18057 Rostock, Germany; 2 Leibniz-Institute for Plasma Science and Technology (INP), Felix-Hausdorff-Str. 2, 17489 Greifswald, Germany

**Keywords:** Chemical surface modifications, Titanium, Plasma polymer, Tissue culture plastic, Collagen type-I, Human osteoblasts, Zeta potential, Cell viability, Signaling, Calcium ion dynamic

## Abstract

**Background:**

Biomaterial modifications—chemical and topographical—are of particular importance for the integration of materials in biosystems. Cells are known to sense these biomaterial characteristics, but it has remained unclear which physiological processes bio modifications trigger. Hence, the question arises of whether the dynamic of intracellular calcium ions is important for the characterization of the cell–material interaction. In our prior research we could demonstrate that a defined geometrical surface topography affects the cell physiology; this was finally detectable in a reduced intracellular calcium mobilization after the addition of adenosine triphosphate (ATP).

**Results:**

This new contribution examines the cell physiology of human osteoblasts concerning the relative cell viability and the calcium ion dynamic on different chemical modifications of silicon–titanium (Ti) substrates. Chemical modifications comprising the coating of Ti surfaces with a plasma polymerized allylamine (PPAAm)-layer or with a thin layer of collagen type-I were compared with a bare Ti substrate as well as tissue culture plastic. For this purpose, the human osteoblasts (MG-63 and primary osteoblasts) were seeded onto the surfaces for 24 h. The relative cell viability was determined by colorimetric measurements of the cell metabolism and relativized to the density of cells quantified using crystal violet staining. The calcium ion dynamic of osteoblasts was evaluated by the calcium imaging analysis of fluo-3 stained vital cells using a confocal laser scanning microscope. The positively charged nano PPAAm-layer resulted in enhanced intracellular calcium ion mobilization after ATP-stimulus and cell viability. This study underlines the importance of the calcium signaling for the manifestation of the cell physiology.

**Conclusions:**

Our current work provides new insights into the intracellular calcium dynamic caused by diverse chemical surface compositions. The calcium ion dynamic appears to be a sensitive parameter for the cell physiology and, thus, may represent a useful approach for evaluating a new biomaterial. In this regard, reliable in vitro-tests of cell behavior at the interface to a material are crucial steps in securing the success of a new biomaterial in medicine.

## Background

Nowadays, there is an increasing demand for permanent, temporary and biodegradable orthopedic devices developed for bone repair and regeneration [1–3]. The cell–biomaterial interaction is a major challenge for tissue engineering. Both the topographical and chemical surface stimuli of the biomaterials can affect cellular behavior, either detrimentally or favorably, at the interface [4–7]. The physico–chemical stimuli of biomaterial surfaces control complex molecular mechanisms responsible for cell function [4, 8–10] by mechanotransduction—translating external signals and forces into intracellular biochemical signals [1]. As a result, initial processes like cell adhesion [8, 11], spreading [9, 12] and the mechanical attachment of cells to the biomaterial surface [5] further influence other cell activities such as proliferation, differentiation [2] and intracellular signaling [4, 10]. There is limited information on whether altered cellular responses by external mechanical stimuli affect intracellular signal transmission via an intracellular calcium ion dynamic. Many cellular functions, like proliferation or differentiation, are regulated by changes of cytosolic free calcium ions (Ca^2+^) [13–15]. The cations (Ca^2+^) act like common intracellular signaling molecules, which function as a “second messenger” [14, 16, 17]. Cytosolic free Ca^2+^-concentration (10^−7^ M) is strictly regulated [16]. A short-term rise of Ca^2+^ is important for signal transmission, and intracellular calcium dynamic is triggered by a variety of factors like adenosine triphosphate (ATP) [14, 17, 18] or mechanical forces [10, 13]. The ligand ATP typically activates the cell-surface G protein-coupled receptor (GPCR) which generates inositol-1,4,5-triphosphate (IP3); this induces transient and rapid Ca^2+^-release through activation of its receptor which is located in the membrane of the internal Ca^2+^-store, the smooth endoplasmic reticulum (ER) [14, 15, 19]. Intracellular Ca^2+^ as a second messenger system is responsible for signal transduction [14] e.g. the transmission of external signals and forces in adaptation to the changed environment [10, 18]. So, external signals provide a distinct Ca^2+^ dynamic that selectively controls long-term cellular responses like proliferation [20] and differentiation [10, 14, 15] by, e.g. binding and activation of other downstream signal proteins and transcription factors [13, 17, 19]. To study the role of the intracellular Ca^2+^ dynamic on different chemical surface compositions, osteoblasts were stained with a very common non-ratiometric (single wavelength) Ca^2+^ indicator fluo-3 [16, 21] and analyzed using confocal laser scanning microscopy. The variation of fluorescence intensity in vital fluo-3-labeled osteoblasts was recorded over the time of 240 cycles of 2 s each [10]. To stimulate the intracellular calcium dynamic, ATP was added after the 90th cycle [10].

**Fig. 1:**
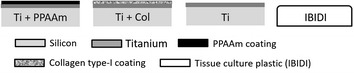
Scheme of the chemical surface composition. The silicon–titanium (Ti) substrates were modified by amino functionalization with plasma polymerized allylamine (Ti* + *PPAAm) as well as immobilization of a bioactive collagen type-I layer (Ti* + *Col). These modifications were compared with bare Ti substrates and tissue culture plastic (IBIDI)

The complex interplay between modified biomaterials and cell behavior has not yet been fully understood and elucidated. Therefore, it is important to determine parameters that reflect the cell physiological behavior of the cells in interaction with the physico–chemical properties of the biomaterial surface. Titanium (Ti) or titanium alloys (like Ti6Al4V) as implant materials in medicine fulfill highly demanding biological conditions, being both inert and biocompatible, having excellent mechanical and physical properties, and being corrosion-resistant [2]. A layer of titanium dioxide (TiO_2_) forms spontaneously when titanium is exposed to air [22]. For an improved interaction of cells on titanium materials, surfaces were endowed with modified chemical as well as physical properties [5, 7, 23]. It is known that cells sense and respond sensitively to the topographical features of surfaces [4]. In this regard, Staehlke et al. [10] found out that osteoblasts on Ti microstructures with impaired cell physiology (cell growth, actin cytoskeleton organization and synthesis of fibronectin) showed significantly reduced intracellular calcium mobilization compared to planar controls. To create new bioactive materials, in addition to the topographic modification, chemical surface properties are of significance for the cell substrate interface [24, 25]. It is reported that the ideal cell adhesion is mediated by positively charged as well as hydrophilic surfaces [2]. The allylamine, polymerized by a low-pressure physical plasma process, generates positively charged amino groups on the wet surface [8, 11]. The advantage of positively charged surfaces is the adsorption of molecules and proteins which mediate cell adhesion [2]. It has been shown that a PPAAm coating causes osteoblasts to respond with, in addition to improved adhesion and increased spreading [7–9], also an improved organization of the actin cytoskeleton with typically long stress fibers and enhanced focal adhesion kinase (FAK) protein expression [11, 12] which finally enhanced cell function [26]. Collagen type-I is one main organic part of the extracellular matrix (ECM), e.g. in skeletal [27] and dental bone [22]. Collagen functions as a ligand for cell adhesion receptors like integrins [6, 10] and is therefore a cell-attractive surface [22]. Collagen type-I layer as a biochemical surface modification supports the cell physiology, including adhesion and differentiation [27, 28]. The objectives of this in vitro-study on human osteoblasts was to investigate the cell physiological effects of two chemically modified Ti surfaces—PPAAm and collagen type-I—compared with a bare Ti substrate, as well as with standard tissue culture plastic (ibiTreat, IBIDI) (see Fig. 1). The zeta potentials on these different chemical compositions of the surface modifications were determined in order to analyze the influence of the surface charge on the cell behavior. Furthermore, this study focused on the intracellular calcium ion dynamic with its importance in the regulation of cell physiology. We have identified the intracellular calcium ion mobilization as a sensitive parameter to observe the cellular behavior on different biomaterials. Reliable in vitro-tests for the description of cell–material interactions are the precondition for the design of new biomaterial surfaces in medicine.

## Results

### Surface characterization reveals a positive surface charge on the PPAAm-nanolayer

**Fig. 2:**
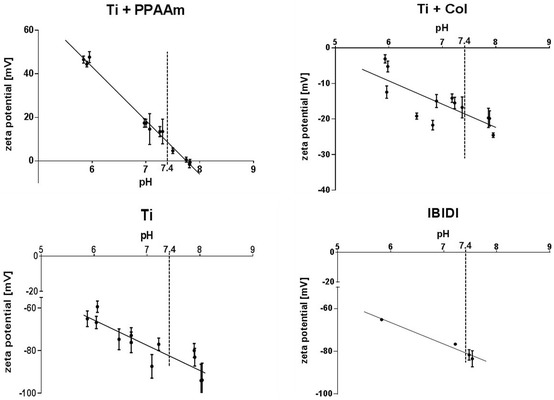
Surface characterization—the zeta potential—of different chemical compositions. Note that the zeta potential of the Ti* + *PPAAm indicated a positive surface charge, whereas collagen type-I coating (Ti* + *Col) and the control surfaces (Ti and IBIDI) exhibited a negative charge. The cell physiological pH 7.4 is indicated. (SurPASS™ system, Anton Paar, n* = *3)

In order to evaluate the existing surface charges of the different chemical surface compositions, the zeta potential at pH 6.0–8.0 was determined (Fig. 2). Table 1 represents the values of the zeta potential at the cell physiological pH 7.4 [7]. These results indicated that only the PPAAm coating on Ti (Ti + PPAAm) exhibits a positive surface charge. In contrast, the immobilized collagen type-I layer on Ti (Ti + Col) revealed a slight negative surface charge, and Ti as well as the tissue culture plastic (IBIDI) surfaces showed a strong negative zeta potential (Table 1).

**Table 1:** Water contact angle and zeta potential (mean ± s.e.m.)

Surface	Water contact angle (in °)	Zeta potential at pH 7.4 (in mV)
Ti	85.0 ± 0.7	− 82.3 ± 3.9 [7]
Ti + PPAAm	68.4 ± 1.7	+ 8.6 ± 1.4 [7]
Ti + Col	60.8 ± 2.2	− 18.5 ± 3.2 [7]
IBIDI	72.8 ± 1.6	− 81.7 ± 2.5

The measurements of water contact angle (WCA, distilled water) indicated that all chemical surface compositions (Ti + PPAAm, 68.4°; Ti + Col, 60.8°) as well as the IBIDI (72.8°) were more hydrophilic compared with Ti (85°) (Table 1).

### The positively charged PPAAm nanolayer displayed increased relative cell viability

**Fig. 3:**
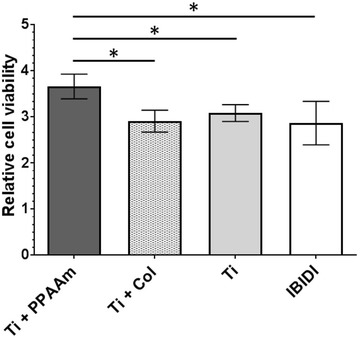
Relative cell viability of MG-63 osteoblasts on the chemical surface compositions after 24 h. Relative cell viability: values from the cell metabolism (MTS) related to the values of the cell density (crystal violet). Note that osteoblasts on Ti* + *PPAAm indicated an increase in relative cell viability compared with Ti* + *Col and bare Ti as well as IBIDI. (Anthos reader, mean* ± *s.e.m., adjusted Mann-Whitney U-Test, *p < 0.05, n* = *5 independent experiments)

The relative cell viability after 24 h was confirmed by colorimetric measurements of the cell metabolism (MTS) and relativized to the density of cells (crystal violet staining). We found a significantly higher relative cell viability of MG-63 cells on Ti + PPAAm (3.66 ± 0.27) in contrast to all negatively charged surfaces after 24 h. A similar cell viability per cell number could be observed for Ti + Col (2.91 ± 0.24), Ti (3.08 ± 0.18) and IBIDI (2.87 ± 0.47) (Fig. 3).

### The positively charged PPAAm nanolayer triggered an enhanced intracellular Ca^2+^ dynamic in MG-63 osteoblasts and HOB

**Fig. 4:**
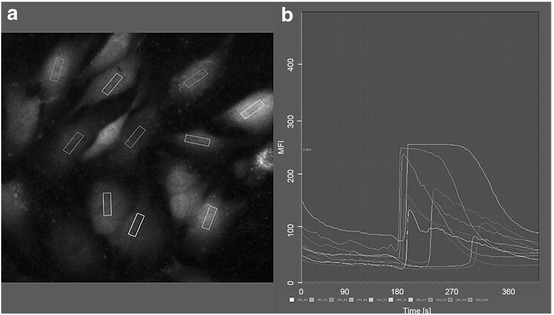
Fluorescence intensity of the Ca^2+^ mobilization in osteoblasts over a time series using confocal laser scanning microscopy (LSM780, Carl Zeiss) and the software ZEN2 (blue edition, Carl Zeiss) in the mode “mean region of interest (ROI)”. **a** In the first fluorescence image of the time series, the defined areas in 10 cells (one area per cell) are fixed. **b** Graphical representation of the mean fluorescence intensity (MFI) of the 10 defined areas over the entire time series

**Fig. 5:**
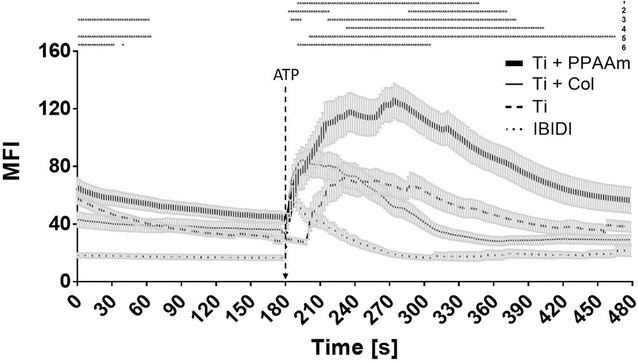
Time courses of Ca^2+^-fluorescence signals of vital fluo-3 loaded MG-63 cells growing on different chemical surface compositions. The addition of ATP is highlighted at the time point 180 s (90th cycle). Note that cells on Ti* + *PPAAm showed significantly increased intracellular calcium signals after ATP stimulation. Cells on the control IBIDI indicated not only a weaker basal calcium signal (without ATP) but also a significantly weaker calcium ion mobilization after ATP compared with osteoblasts on Ti, Ti* + *PPAAm, and Ti* + *Col. (LSM780, Carl Zeiss; 3 independent approaches for 10 defined areas each of 10 cells per time point, polygon line as the mean* ± *s.e.m., multiple t-test, *p < 0.05). Explanations: 1* = *Ti vs. Ti* + *PPAAm, 2* = *Ti vs. Ti* + *Col, 3* = *Ti vs. IBIDI, 4* = *Ti* + *PPAAm vs. Ti* + *Col, 5* = *Ti* + *PPAAm vs. IBIDI, 6* = *Ti* + *Col vs. IBIDI

Fluorescence measurements of vital fluo-3/acetoxymethyl ester (AM)-stained osteoblasts were recorded on the confocal laser scanning microscope (LSM780). The mean fluorescence intensity (MFI) of 10 defined regions of the cells (one region per cell, see Fig. 4) was analyzed per cycle over a time series (240 cycles every 2 s, total 480 s). To stimulate the cytoplasmic Ca^2+^ rise from the endoplasmic reticulum (ER), ATP was added after the 90th cycle (180 s). The recorded fluorescence signal of the stained cells over time was evaluated as a (i) basal calcium level (without ATP stimulation, 0–180 s), and (ii) the calcium ion mobilization (after ATP stimulation, 182–480 s). The individual values can be found in Table 2. MG-63 cells on Ti + PPAAm showed a significantly increased intracellular Ca^2+^-mobilization after stimulation with ATP in contrast to Ti + Col, Ti and IBIDI (Fig. 5). The results indicated that the Ca^2+^ dynamic in MG-63 osteoblasts was influenced by a positively charged surface.

**Table 2:** Mean fluorescence intensity of mobilized Ca^2+^ in MG-63 cells on Ti substrates (mean ± s.e.m.)

Ca^2+^ signal	Ti	Ti + PPAAm	Ti + Col	IBIDI
Basal level (0–180 s)	38.7 ± 0.8	51.9 ± 0.6	38.6 ± 0.2	23.6 ± 0.8
After ATP (182–480 s)	49.9 ± 1.1	89.1 ± 1.9	44.8 ± 1.6	17.2 ± 0.1

It has often been discussed that the tumor cell lines (e.g. the MG-63 cells) are different in its sensitivity to primary cells.

**Fig. 6:**
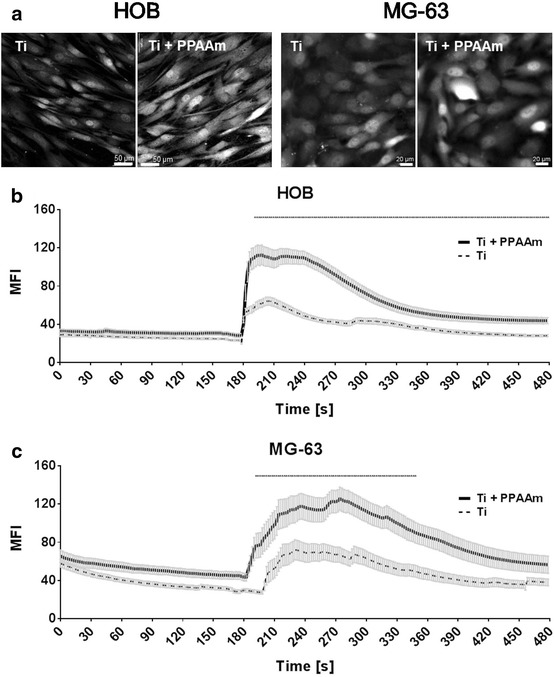
Calcium imaging in vital fluo-3 loaded human osteoblasts—cell line (MG-63) and primary osteoblasts (HOB). Changes in fluorescence intensities of the cells were detected using confocal laser scanning microscopy (LSM780, Carl Zeiss, ZEN-software). **a** The representative fluorescence images, after ATP stimulation (240 s* = *120th cycle), show the Ca^2+^ increase on Ti* + *PPAAm surfaces compared with bare Ti in MG-63 as well as HOB cells. (Scale bar MG-63: 20 µm, HOB: 50 µm). **b** Intracellular Ca^2+^ dynamics in vital primary osteoblasts (HOB) over 480 s (= 240 cycles) every 2 s on Ti* + *PPAAm compared with Ti. Note the significantly enhanced intracellular calcium ion signal in HOB cells after ATP stimulus (180 s) on Ti* + *PPAAm. **c** Intracellular Ca^2+^ signal of MG-63 cells over time on Ti* + *PPAAm compared with Ti. Significant differences in the intracellular Ca^2+^ dynamic of MG-63 after ATP stimulation was detectable (see explanation in **b**). MG-63 and HOB established the same cellular reactivity in calcium signaling on Ti* + *PPAAm and bare Ti. (MG-63* = *3, and HOB* = *5 independent approaches each for 10 defined areas of 10 cells per time point, 240 cycles, polygon line as mean* ± *s.e.m., multiple t-test, *p < 0.05)

In order to confirm the influence of chemical modifications on intracellular Ca^2+^ signaling also in human primary osteoblasts (HOB), experiments were done in direct comparison. MG-63 and HOB cells were cultured for 24 h only on the most noticeable chemical modification, the positively charged Ti + PPAAm, compared with bare Ti. The fluorescence intensity of these fluo-3-stained osteoblasts at the 120th cycle was higher in both HOB and MG-63 cells on Ti + PPAAm (Fig. 6a). A significantly increased cytosolic free Ca^2+^ mobilization after ATP stimulation was found in HOB as well as in MG-63 cells on Ti + PPAAm compared with Ti (Fig. 6b, c). The fluorescence signals are indicated in Table 3. Thus, the primary osteoblasts confirm increased calcium signaling on Ti + PPAAm.

**Table 3:** Comparison of Ca^2+^ signals in HOB and MG-63 cells on Ti and Ti + PPAAm (mean ± s.e.m.)

Ca^2+^ signal	Primary osteoblasts—HOB		Osteoblast cell line—MG-63	
	Ti	Ti + PPAAm	Ti	Ti + PPAAm
Basal level (0–180 s)	26.3 ± 0.3	31.2 ± 0.1	38.7 ± 0.8	51.9 ± 0.6
ATP peak maximum (182–300 s)	50.5 ± 1.0	99.2 ± 1.7*	59.3 ± 1.9	106.6 ± 2.5*
Slope after ATP (302–480 s)	33.4 ± 0.5	50.3 ± 0.8*	43.8 ± 0.8	77.8 ± 1.8

* Statistically significant, p < 0.05, multiple *t*-test

## Discussion

In the present study, osteoblast behavior (viability and calcium signaling) was correlated to different chemical surface compositions on titanium—plasma polymerized allylamine (Ti + PPAAm) and a collagen type-I coating (Ti + Col)—in comparison with the bare substrate (Ti) and tissue culture plastic (IBIDI). Using in vitro approaches, we showed clearly that chemical surface modifications of biomaterials affect the relative cell viability and, further, the intracellular Ca^2+^ dynamic in osteoblasts. In addition, we found a biological analysis method—calcium imaging—to determine cell regulatory mechanisms which reflect the cell behavior on different materials.

Titanium is the biomaterial of choice in medical devices due to its mechanical and biological compatibility [22, 28]. Today, new biomaterials in medicine should be biocompatible and furthermore actively promote cellular functions [2]. Bioactive material surfaces are developed by physico–chemical modification for better osseointegration [4]. Different studies investigated the influence of surface modifications on cell–material interaction [4, 5]. The research and evaluation of new materials requires additional in vitro tests, also for the assessment of signal transduction.

The interaction between materials and osteoblasts is dependent on surface characteristics like wettability, surface charge or surface energy [3]. First of all, we analyzed the surface charge (at pH 7.4) of the different chemical compositions [7]. The zeta potential is of importance for biological responses like adhesion and spreading [4, 8, 9]. The zeta potential of surfaces modified with a Ti + PPAAm showed the only positive surface charge of all surfaces used in the study. The other chemical titanium modification Ti + Col indicated a slight negative surface charge. Our comparative surfaces—bare Ti substrate as well as IBIDI—revealed a highly negative zeta potential [7].

Previous studies, which characterized the PPAAm coating, examined the positive zeta potential in addition to hydrophilicity of this nanolayer [8, 11]. Interestingly, a greater hydrophilic potential was also observed for a collagen type-I coating, but in contrast to the PPAAm-nanolayer, a negative surface charge was measured [7]. Rebl et al. [9] reported a negative surface charge for a collagen type-I layer on glass as well. Likewise, IBIDI dishes, with their high negative charge, exhibit hydrophilic properties [29]. It is known that biomaterial surfaces with moderate hydrophilicity improve cell growth and biocompatibility [2]. In addition, improved initial cell adhesion on positively charged surfaces was previously observed [7–9]. The extracellular matrix (ECM) molecules, which are synthesized by osteoblasts and important for cell adhesion and regeneration [22], are negatively charged, e.g. hyaluronan or collagen type-I [12]. The collagen type-I is, on the basis of the abundant presence in human tissue, the stability and as part of the ECM, a potential, organic bioactive coating for titanium surfaces [22]. The positively charged PPAAm nanolayer is capable of attracting negatively charged biomolecules of the ECM [12], and therefore particularly suitable as a coating for biofunctionalized implant surfaces. Anselme et al. [4] described the electric charge, existing at the interface of biomaterials, as a significant factor in protein adsorption and integrin binding. It is postulated that for the best cell attachment a positively charged surface should be used, since cell adhesion and spreading are influenced by chemical composition and surface potential [2]. The initial cell reaction controls further cellular responses like signaling events and finally cell viability [2]. Major regulators of cell viability and proliferation are cell adhesion and cell spreading [5]. Prior investigations on surfaces coated with PPAAm indicated a significant increase in initial osteoblast adhesion and spreading [9, 11, 12], and thus an enhanced cell–material contact which finally improved the proliferation rate [26]. The positively charged modification with a nanolayer of PPAAm seems to be attractive for cells and stabilizes the adhesion of cells on the biomaterial surface, resulting, in addition, in better implant osseointegration [8, 12, 26].

The new findings of this study confirm this assumption. In this study, osteoblasts which were cultured for 24 h on surfaces with a positively charged PPAAm nanolayer showed a significant increase of relative cell viability compared with negatively charged surfaces. Cell viability on Ti + Col appears to be unchanged compared with the Ti and IBIDI. It is postulated in the literature that a bioactive protein coating with collagen type-I acts as a ligand for cell adhesion receptors [4, 6] and therefore will be a cell attractive surface [25]. In this regard, in in vitro- and in vivo-studies Avila et al. [22] presented enhanced cellular behavior on collagen type-I coated implants. Also, in long-term experiments (8 weeks) Sverzut et al. [25] showed that collagen type-I coatings led to improved osseointegration and differentiation in vivo. In the proximal tibial metaphysis of rats, Reyes et al. [30] compared bone-to-implant contact of machined titanium surfaces (Ti) with e.g. bovine type I collagen (Col I). After 4 weeks of healing the mean bone-to-implant contact percentages were 58% for Col I and 43% for Ti. Col I was statistically higher compared with Ti. The authors concluded that Col I enhanced bone repair and implant integration.

Further literature showed inconsistent results in in vitro approaches. Also, Morra et al. [28] postulated that the osteoblast growth rate on collagen-modified biomaterials is lower and cell viability is similar compared with pure titanium. Regarding our study, Rebl et al. [9] were also able to show that the positive charges of a PPAAm coating enhanced the cell physiology (initial cell adhesion and spreading), and were more effective than the collagen type-I coated surfaces. Thus, the zeta potential seems to be an important surface characteristic for cell physiology, as demonstrated in this study with the relative cell viability.

This feature of cell physiology—relative cell viability—is also reflected in the intracellular calcium dynamic after stimulation with ATP. In this study, an adapted cytosolic calcium signal was found corresponding to the relative cell viability in MG-63 osteoblasts on the different chemical compositions. It is known from the literature that intracellular Ca^2+^ is a component of downstream signaling cascades [13, 14, 17] and regulates characteristics of cell physiology such as proliferation [15, 20]. The analysis of the intracellular Ca^2+^ dynamic in fluo-3 stained osteoblasts on different chemical surface compositions presented here were performed using confocal laser scanning microscopy. The fluorescence changes over the time of 240 cycles were recorded every 2 s and evaluated. The basal calcium level (without stimulation), and the calcium dynamic after stimulation with ATP (even at the 90th cycle) were determined [10]. Interestingly, the cells on positively charged PPAAm reacted with a significantly higher calcium signal after ATP-stimulus compared with cells on negatively charged surfaces. Some studies showed a similar cellular reaction of the cell line MG-63 and primary osteoblasts (HOB) on biomaterials concerning the expression of integrin subunits and signaling, and declared MG-63 cells to be useful in in vitro models [31]. Nevertheless, they intend to verify appropriate functional studies with human primary cells [31]. Therefore, we placed HOB on the most noticeable chemical modification (Ti + PPAAm) compared with bare Ti and analyzed the intracellular calcium signal. Not only in the MG-63 cell line, but also in HOB, we were able to demonstrate this phenomenon of increased Ca^2+^ mobilization on Ti + PPAAm. Also, Ravenscroft et al. [24] showed that chemical surface features of self-assembled monolayers on coverslips (DETA) can influence the calcium dynamic after an electrical stimulation (1 HZ, 6 V signal, 5 ms per pulse), as demonstrated with fura 2-stained chicken embryonic cardiac myocytes. Cells on the hydrophilic silanes showed a significant higher excitation-induced Ca^2+^ concentration and dynamic (i.e. calcium transients, amplitude and duration).

Due to good spreading and adhesion properties, adhesion receptors such as integrins mediated stronger calcium signals [13]. Furthermore, cytoskeletal organization and formation of a cytoskeletal signaling complex affects the intracellular calcium mobilization [13]. In previous studies it was found that osteoblasts grown on the PPAAm nanolayer displayed improved osteoblast adhesion and spreading as well as a strong actin filament network [11, 12]. This new, further study shows accordingly the significantly increased calcium ion dynamic in osteoblasts on this positively charged surface.

In another earlier study we indicated altered cellular behavior on the topography of a defined micro-structured surface (micro pillars): changed actin organization resulting in short fiber formation on the top of the pillars [10] and, as a consequence, a significant decrease in the intracellular calcium signal [10, 16]. These topography-dependent reactions finally led to inhibited cell function [10]. Thus, we suggest that osteoblasts transmitted external signals and forces from the environment into the cell via calcium signaling. The stringent regulation of the intracellular Ca^2+^ dynamic plays an important role in cell function [10, 13, 15]. It seems to be a correlation between the increased viability of MG-63 cells and the enhanced calcium ion dynamic detected on Ti + PPAAm. In this regard, it has been shown that the intracellular Ca^2+^ dynamic plays an important role, and thus can be an indicator for the behavior of cells on different biomaterials.

## Conclusion

The material surface functionalization with positively charged plasma-polymerized allylamine (PPAAm) resulted in increased cell viability and, furthermore, in an enhanced calcium ion mobilization after ATP stimulation. We conclude that the calcium ion dynamic reflects the behavior of the cells on different surfaces accordingly. To develop new biomaterials it is of importance to understand the interaction of cells with the underlying material.

We have found an in vitro-method—calcium imaging—to assess the cell response to functionalized surface modifications. This study is one of the first to examine the intracellular calcium ion level and stimulus-dependent dynamic of intracellular calcium ions on chemically different coatings. These new results support our hypothesis that the calcium ion dynamic in cells is important in the transmission of external signals into the cell, which finally regulate the cell physiology.

## Methods

### Surfaces and chemical composition

The bare substrate was silicon with a final 100 nm titanium (Ti) coating. The surfaces thus had a native titanium-oxide layer (TiO_2_) at which the cells interact. The Ti wafers, which measured 1 × 1 cm (length x width), were obtained from the Center for Microtechnologies (ZFM, University of Technology Chemnitz, Germany). To sterilize the bare material, samples were incubated in 70% ethanol for 15 min and rinsed with phosphate buffer saline (PBS, Sigma Aldrich, Munich, Germany).

One of the modifications of the bare Ti substrate was the wet chemical coating with collagen type-I (Ti + Col). For this purpose, 200 µl of a collagen work solution (Col, type I, rat tail tendon, BD Bioscience, Heidelberg, Germany, 200 µg/ml in acetic acid) was dripped onto the Ti sample under sterile conditions (laminar flow box) and allowed to adhere for 3 h. To remove the acetic acid from the working solution, the surfaces were rinsed 3 times with PBS before use.

Another chemical modification of the bare Ti was the coating with plasma polymerized allylamine (PPAAm) (Ti + PPAAm). The preparation was carried out in a low-pressure microwave plasma reactor (2.45 GHz; 500 W, 50 Pa) V55G (Plasma Finish, Germany, V = 60 l). The samples underwent a two-step procedure: at first they were decontaminated and activated in pulsed oxygen plasma (10 ms on/90 ms off; 30 s effective) and then, without breaking the vacuum, coated with the monomer allylamine by the pulsed plasma polymerization process (300 ms on, 1700 ms off, 72 s effective, gross 480 s). Prior to use, the allylamine was carefully purified of air by evacuating and purging with N_2_. A liquid handling system allowed for the exact dosing of allylamine. Argon was used as a carrier gas (50 sccm Ar). The substrate was located in the plasma reactor in a downstream position [11]. The thickness of the deposited coatings was around 25 nm. Before the experiments started, these surfaces were rinsed with PBS.

The following control surfaces were used for the experiments—a bare Ti wafer (see above) and a tissue culture plastic (IBIDI). The tissue culture plastic ibidi µ-dishes with polymer coverslip (ibiTreat, Ø 35 mm; ibidi GmbH, Martinsried, Germany) are appropriate for microscopy and cell-based assays and fulfill optical requirements [29].

### Surface characterization—surface charge

Zeta-potential measurements were performed using the SurPASS™ system (Anton Paar, Ostfildern, Germany) to determine the surface charge. Smooth samples with a size of 2 × 1 cm were mounted pairwise in the chamber with a gap height of 100 µm. The measurements were performed in a 0.001 mol/l KCl solution ranging from pH 6.0 to 8.0. The streaming current was determined depending on the pressure (max. 400 mbar). Finally, the zeta potential was calculated according to the method of Helmholtz–Smoluchowski. Measurements were performed in quadruplicate on three independent pairs of samples (except for IBIDI, where only two samples were measured).

### Surface characterization—wettability

The water contact angle (WCA) was determined by the sessile drop method using the Drop Shape Analyzer—DSA25 (Krüss GmbH, Hamburg, Germany) and 2 μl distilled water. Drop images were acquired with the digital camera of the DSA25, and the contact angles were determined with the included software by the fit method ellipse (ADVANCE, V.1.7.2.1). The measurements were done with three sessile drops on one sample (technical triplicates), and 3 independent samples were used for each surface (n = 3).

### Cell culture

For the main part of the experiments, MG-63 cells, a human osteoblast-like cell line from ATCC (American Type Culture Collection ATCC^®^, CRL1427™) were used. The MG-63 cell line has similar characteristics concerning morphological behavior, adhesion, integrin receptor expression and signaling properties to primary human osteoblasts [31]. For comparison and confirmation of the intracellular calcium ion dynamic we used human primary osteoblasts (HOB, PromoCell GmbH, Heidelberg, Germany, C-12720) as well. Both cell types were cultured at 37 °C in a humidified atmosphere (5% CO_2_). MG-63 cells were grown in Dulbecco’s modified eagle medium (DMEM; Life Technologies GmbH, Darmstadt, Germany), with 10% fetal calf serum (FCS, Biochrom FCS Superior, Merck KGaA, Darmstadt, Germany) and 1% antibiotic (gentamicin, Ratiopharm GmbH, Ulm, Germany). Experiments were performed in passages 5–25. The HOB cells were cultivated in osteoblast growth medium with SupplementMix (PromoCell) and 1% antibiotic–antimycotic (Anti-Anti 100×, Life Technologies). Investigations with HOB were done in low passages (two to four). All cell cultures were examined prior to further examination on the one hand for mycoplasma, and on the other hand for density and growth. Cells in the near-confluent state (70–80% of confluency) were used for the corresponding in vitro-experiments.

### Relative cell viability assay

To estimate the relative viability of cells growing on different chemical surface compositions, MTS assay was used. MTS ((3-(4,5-dimethylthiazol-2-yl)-5-(3-carboxymethoxyphenyl)-2-(4-sulfophenyl)-2*H*-tetrazolium salt), a yellow tetrazolium, is reduced to purple formazan in living cells by mitochondrial metabolic activity. The absorbance of this colored solution could be measured. For this, MG-63 cells (50,000 cells/cm^2^) were seeded onto the 1 × 1 cm surfaces in 24-well plates (Thermo Fisher Scientific, Roskilde, Denmark) and cultivated for 24 h. Thereafter, materials with adherent cells were transferred into a fresh 24-well plate. Here, cells were incubated for 2–3 h in 500 µl culture medium containing 100 µl of MTS reagent (CellTiter 96^®^ Aqueous ONE-Solution Cell Proliferation Assay, Promega, USA) at 37 °C. Supernatants were transferred into a 96-well plate (for each experimental group 4 × 100 µl were analyzed). The absorbance was recorded at 490 nm with a microplate reader (Anthos, Mikrosysteme, Krefeld, Germany). A background measurement was taken at 650 nm. To quantify the cell number, crystal violet staining was performed. Crystal violet binds to the negatively charged DNA in a linear fashion via ionic attraction. Staining was done on the basis of a protocol published previously [32]. Briefly, cells were fixed in 2-propanol (Walter CMP GmbH, Kiel, Germany) after washing with PBS. The permeabilization of the cell membrane was achieved by washing with 0.05% Tween 20 (VWR Chemicals, Leuven, Belgium). After shaking with 0.1% crystal violet solution (Serva, Heidelberg, Germany) for 20 min at room temperature, cells were washed with double-distilled (dd) H_2_O. The bound crystal violet was re-dissolved using 33% acetic acid (J. T. Baker, Deventer, Netherlands). The optical density of the transferred supernatants was quantified with a microplate reader at 620 nm. The relative cell viability of osteoblasts will be presented as a quotient of MTS and crystal violet data.

### Intracellular Ca^2+^ dynamic analysis by calcium imaging

For the live cell calcium imaging, 80,000 cells/cm^2^ osteoblasts were cultured on 1 × 1 cm samples with different chemical compositions for 24 h, and afterwards washed with pre-warmed PBS (+ Ca/Mg, Sigma) and stained with the calcium indicator fluo-3/AM (Life Technologies Corporation, Eugene, Oregon, US, 5 µM) according to Staehlke et al. [10]. Briefly, cells were transferred to a slightly hypotonic 4-(2-hydroxyethyl)-1-piperazineethanesulfonic acid (HEPES) buffer and loaded with fluo-3/AM. Fluo-3 is engineered with acetoxymethyl (AM) ester to load the dye into the osteoblasts [21]. In order to completely incorporate the dye in the osteoblasts for the best fluorescence signal, the method of hypo-osmotic shock treatment was additionally applied [16]. After incubation of the fluo-3/AM (40 min at 37 °C), the cells were cultured further in an isotonic HEPES buffer. Vital fluo-3/AM-labeled osteoblasts were visualized by a confocal laser scanning microscope (LSM780, Carl Zeiss AG, Oberkochen, Germany) with a C Apochromat 40× water immersion objective (Carl Zeiss, 1.20 W Korr M27) and an excitation at 488 nm by the argon ion laser (emission at 515 nm). To record the global Ca^2+^ fluorescence signal from single cells, the mode “time series” of the ZEN software (ZEISS efficient navigation, ZEN 2011 SP4, black edition, Carl Zeiss) of one cycle every 2 s for 240 cycles was applied. In order to stimulate the intracellular Ca^2+^ release from the endoplasmic reticulum and thus the intracellular calcium dynamic, ATP (adenosine 50-triphosphate, 10 µM, SERVA Electrophoresis GmbH, Heidelberg, Germany) was added to the experiment always at the same time point—after the 90th cycle—during the recording of the time series. At least three independent samples were analyzed for each experimental group to assess the chemical surface influence on the calcium ion dynamic. Samples were exposed with the same settings (Gain, Digital Offset) as well as with a pinhole of maximal airy units (15 AU, 13.5 µm section). The measurement of the mean fluorescence intensity (MFI) of the global Ca^2+^ signal from the separate images of the time series was done by ZEN2 (blue edition, version 2.0.0.0, Carl Zeiss). Ten defined areas of cells (one area per cell) for each time point (240 cycles = 240 time points) were analyzed using the function “mean ROI” (mean region of interest). For this, the corresponding defined areas were selected in the first image of a time series and the software analyzed the mean fluorescence intensity (MFI) of these areas in each cycle (one cycle = one image) of the entire time series (Fig. 4). Fluorescence images were acquired at a resolution of 512 × 512 pixels.

### Statistical evaluation

The statistical evaluation was conducted at least three times in independent tests. Results for the in vitro-investigations are expressed as mean ± standard error of the mean (s.e.m.). For the relative cell viability, we used the Mann–Whitney U-test. For the intracellular Ca^2+^ dynamic experiments, a multiple *t*-test was done. Significant differences were reported as adjusted p-values < 0.05 (two-sided). All statistical analyses were performed with GraphPad Prism7 software (GraphPad Software Inc., La Jolla, CA USA).

## Abbreviations

AM: acetoxymethyl ester
ATCC: American type culture collection
ATP: adenosine triphosphate
AU: airy unit
Ca^2+^: calcium ions
Col: collagen type-I
DMEM: Dulbecco’s modified eagle medium
ER: endoplasmic reticulum
ECM: extracellular matrix
FACS: fluorescence-activated cell scanning
FCS: fetal calf serum
HEPES: 4-(2-hydroxyethyl)-1-piperazine-ethanesulfonic acid
HOB: human primary osteoblasts (PromoCell)
ibidi: Integrated BioDiagnostics (tissue culture plastic)
LSM: laser scanning microscope
MFI: mean fluorescence intensity
MTS: 3-(4,5-dimethylthiazol-2-yl)-5-(3-carboxymethoxyphenyl)-2-(4-sulfophenyl)-2*H*-tetrazolium salt
PBS: phosphate buffered saline
PPAAm: plasma polymerized allylamine
s.e.m.: standard error of the mean
Si: silicon
Ti: titanium
WCA: water contact angle
ZEN: ZEISS efficient navigation
